# Upper airway obstruction during sleep in infants with laryngomalacia is frequently sleep-position-dependent

**DOI:** 10.1038/s41390-025-03919-z

**Published:** 2025-02-13

**Authors:** Turkka Kirjavainen, Mervi Kanerva, Hanna-Leena Kukkola, Johanna Nokso-Koivisto

**Affiliations:** 1 Department of Pediatrics, New Children’s Hospital, Helsinki, Finland; 2 Pediatric Research Center, New Children’s Hospital, University of Helsinki, Helsinki, Finland; 3 Children’s Hospital Department of Clinical Neurophysiology and Neurological Sciences, HUS Medical Imaging Center, Helsinki University Hospital, Helsinki, Finland; 4 Department of Otorhinolaryngology, Head and Neck Surgery, Helsinki University Hospital, Helsinki, Finland

## Abstract

**Background:**

Laryngomalacia is the most common congenital airway anomaly causing breathing difficulties in infants. Severe laryngomalacia is often associated with obstructive sleep apnea (OSA).

**Methods:**

We re-evaluated 14-year pediatric sleep center polysomnography (PSG) data in infants with fluoroscopy-verified laryngomalacia.

**Results:**

The study included 79 infants, with a median corrected age of 8 weeks (interquartile range, IQR 5–13) and a laryngomalacia clinical score of 10/14 (IQR 7–11). Most (78%) PSG studies were daytime studies. In PSG, laryngomalacia-related breathing difficulty appeared as a sleep stage and position-dependent OSA with laborious breathing. PSG allowed position comparison in 69 infants. In the supine sleeping position, a median obstructive apnea and hypopnea-index (OAHI) was 22 h^−1^ (IQR 10–50) compared with 7 h^−1^ (IQR 1–26) in the side sleeping position (*p* < 0.0001). In the supine, breathing was also more laborious, and end-tidal carbon dioxide 99th percentile levels were higher than in the side sleeping position (*p* < 0.0001). The degree of OSA (OAHI) showed only a weak correlation with the laryngoscopy severity score (*R*^2^ 0.10, *p* = 0.005).

**Conclusions:**

In infant laryngomalacia, the degree of upper airway obstruction is frequently more severe in the supine than in the side sleeping position. However, some variability remains in the response.

**Impact:**

- Laryngomalacia is the most common congenital airway anomaly causing breathing difficulties in infants. Obstructive breathing events and obstructive sleep apnea are common in severe laryngomalacia even though the stridor often diminishes or resolves during sleep.
- We observed that in young infants with laryngomalacia, the appearance of upper airway obstruction is both sleep position and sleep-stage dependent.
- Compared to the supine sleeping position, the side sleeping position reduced the frequency of obstructive events and breathing effort, and lowered end-tidal carbon dioxide 99th percentile levels.

## Introduction

Laryngomalacia is the most common congenital airway anomaly causing breathing difficulties in infants.^1^ In most cases, respiratory symptoms occur during the first few weeks of life, and may worsen over the first four to eight months before resolution by the age of 18–24 months.^2^ Respiratory stridor and breathing difficulties are exacerbated by agitation, crying, feeding, upper respiratory tract infections, or supine positioning.^1^ Obstructive breathing events and obstructive sleep apnea (OSA) are common even though the stridor often diminishes or resolves during sleep.^3–5^ Recognition of sleep-related breathing disorders in infants with laryngomalacia has increased the use of high nasal flow cannula and positive airway pressure (CPAP) treatment together with surgery such as supraglottoplasty in the management of laryngomalacia.^4^ In laryngomalacia, the recovery of OSA may be slow, occurring gradually by the age of three years.^6^

In infants, OSA is commonly sleep position dependent.^7–10^ After recognition of sleep position dependency of OSA in most infants,^7–10^ we applied supine and side sleep position testing on polysomnography protocol in all infants with suspected upper airway obstruction. In this study, we evaluated the effect of sleep positioning on upper airway obstructions in young infants diagnosed with moderate or severe laryngomalacia. The results were compared to two historical groups: infants with Robin sequence (PRS)^10^ and infants with OSA without any obvious predisposing anatomical factors.^8^

## Methods

### Study design and patients

We evaluated New Children’s Hospital pediatric sleep unit data between November 2010 to April 2024. Infants less than nine months with observed laryngomalacia, and with both flexible laryngeal fiberoscopy and polysomnography (PSG) evaluation were included (Table 1). PSG results were compared to those of 123 infants with PRS^10^ and 77 infants with OSA without any obvious anatomical predisposing factors.^8^ All infant groups were studied using the same study protocol. The local pediatric ethics committee (HUS/3186/2019) and the New Children’s Hospital Institutional Review Board (Project #4964) approved the study protocol.

**Table 1:** Demographic data.

	All infants	Infants included in sleep position comparison
Number of infants	79	69
Born prematurely [*n* (%)]	10 (13%)	7 (10%)
Corrected age [weeks (IQR)]	8.1 (4.7–13.4)	8.1 (4.7–12.9)
Female [*n* (%)]	33 (42%)	32 (46%)
Male [*n* (%)]	46 (58%)	37 (54%)
Co-existing predisposing factor for OSA	39 (49%)	33 (48%)
Gastroesophageal reflux symptoms [*n* (%)]	26 (33%)	24 (35%)
Small chin [*n* (%)]	7 (9%)	7 (10%)
Nasal airway [*n* (%)]	2 (3%)	2 (3%)
Left-sided vocal cord palsy [*n* (%)]	1 (1%)	1 (1%)
Down syndrome [*n* (%)]	2 (3%)	1 (1%)
Chromosome deletion [*n* (%)]	2 (3%)	1 (1%)
Miller-Dieker syndrome [*n* (%)]	1 (1%)	1 (1%)
Plagiocephalia [*n* (%)]	1 (1%)	0 (0%)
Growth failure (1–12 months)		
Minimum weight percentage [median % (IQR)]	−9.0 (−15.1 to −3.0)	−9.0 (−13.9 to −3.0)
Minimum weight percentage < −10% [*n* (%)]	32 (41%)	29 (42%)
Minimum weight percentage < −15% [*n* (%)]	19 (24%)	15 (22%)

Results are presented as a number of infants (percent) or median (IQR, interquartile range).

Weight percentage represents the calculated percentual change of weight against the predicted normal mean weight of infants with the same height.^13^

### Polysomnography

We performed sleep studies in the supine and side positions if obstructive events were suspected or observed during online PSG analysis. Infants were also placed in a prone position on a few occasions. In each position, we recorded at least one cycle of rapid-eye-movement sleep (REM) and of non-REM sleep. These sleep studies took place during daytime in infants less than three months old, and during night-time in older infants. The majority of PSG were done as daytime recordings (Table 2).

**Table 2:** Characteristics of laryngomalacia, videoscopy, and polysomnography.

	All infants	Infants included in sleep position comparison
Number of infants	79	69
Videoscopy		
Videolaryngoscopy [mean (SD)]	2.6 (SD 1.9)	2.6 (SD 1.9)
Wake [*n* (%)]	54 (68%)	50 (72%)
Sleep [*n* (%)]	10 (13%)	7 (10%)
Wake and sleep	15 (19%)	12 (17%)
Upright [*n* (%)]	51 (65%)	47 (68%)
Supine [n (%)]	11 (14%)	8 (12%)
Upright and supine [*n* (%)]	17 (22%)	14 (20%)
Video reanalyzed	37 (47%)	35 (51%)
Laryngomalacia severity		
Clinical score	10 (7–11)/14	10 (7–11)/14)
History score [median (IQR)]	7 (IQR 5–8)/10	7 (5–8)/10
Physical examination [median (IQR)]	3 (IQR 2–3)/4	3 (2–3)/4
Videolaryngoscopic score [median (IQR)]	4 (IQR 3–5)/8	4 (4–6)/8
Arythenoid score [median (IQR)]	2 (IQR 2–3)/4	2 (2–3)/4
Epiglottic score [median (IQR)]	2 (IQR 1–3)/4	2 (1–3)/4
Laryngomalacia type		
Type 1 [*n* (%)]	32 (41%)	27 (39%)
Type 2 [*n* (%)]	41 (52%)	36 (52%)
Type 3 [*n* (%)]	6 (8%)	6 (9%)
OAHI in Type 1 (h^−^^1^) [median (IQR)]	21.5 (IQR 10.9–34.9)	21.5 (IQR13.3–34.9)
OAHI in Type 2 (h^−1^) [median (IQR)]	14.3 (IQR 3.9–40.5)	16.2 (IQR 4.5–45.6)
OAHI in Type 3 (h^−1^) [median (IQR)]	74.2 (IQR 57.6–102.1)	74.2 (IQR 57.6–102.1)
Polysomography (PSG)		
Number of performed PSGs [mean (SD)]	1.6 (SD 1.0)	1.6 (SD 1.1)
Characteristics of included PSG		
Daytime nap-study [*n* (%)]	62 (78%)	56 (81%)
Overnight study [*n* (%)]	17 (22%)	13 (19%)
Recording time (min) [median (IQR)]	228 (196–312)	228 (196–300)
Sleep characteristics		
Total sleep time (min)	150 (128–198)	148 (129–195)
Sleep efficiency [% (IQR)]	70 (59–76)	70 (60–76)
Time in non-REM sleep (min)	102 (85–136)	102 (87–131)
Time in REM sleep (min)	50 (37–71)	47 (39–66)
Time in REM sleep/total sleep time (%)	30 (24–38)	30 (24–38)

Results are presented either as median (IQR, interquartile range) or mean (SD, standard deviation).

**Fig. 1:**
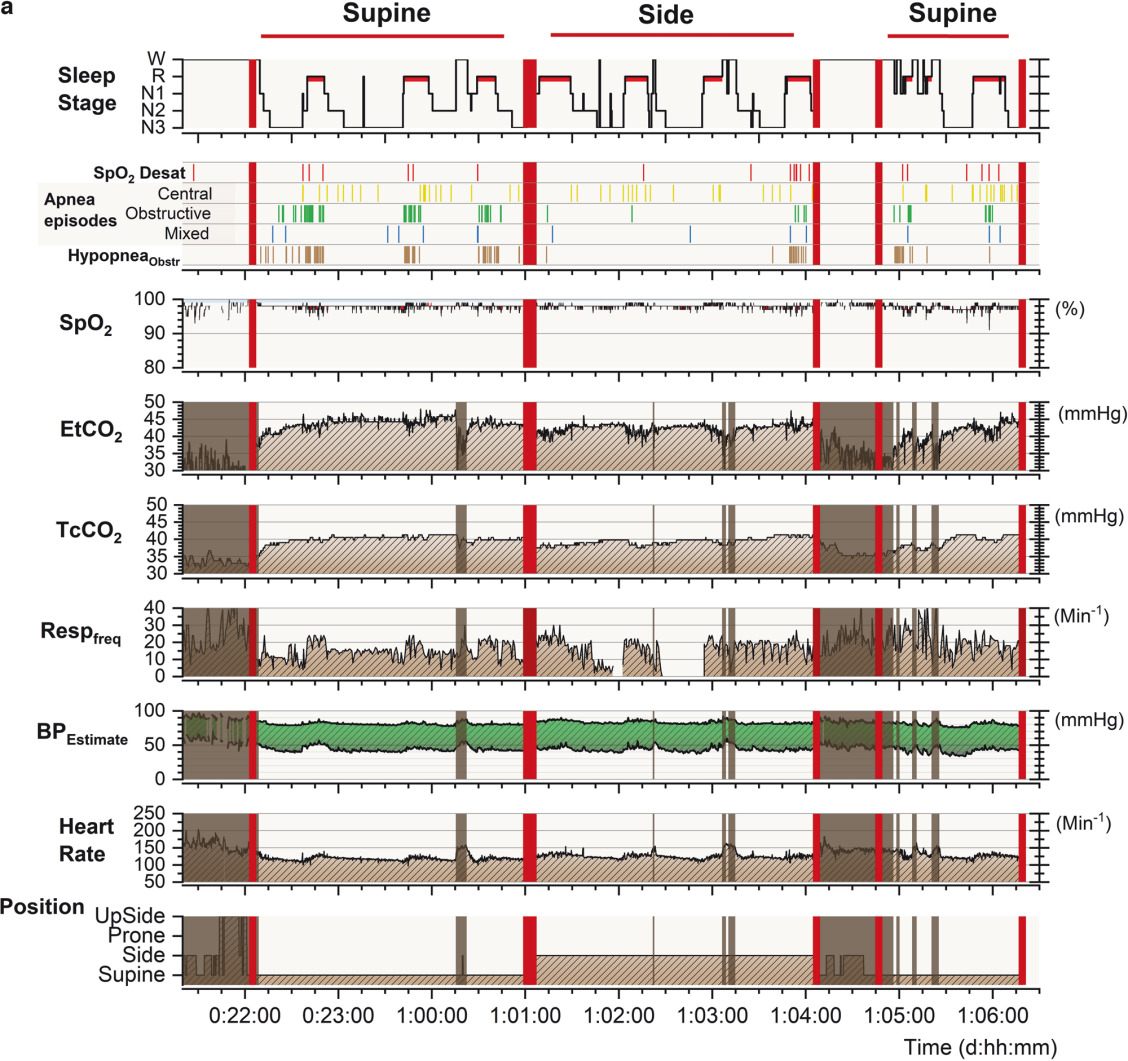

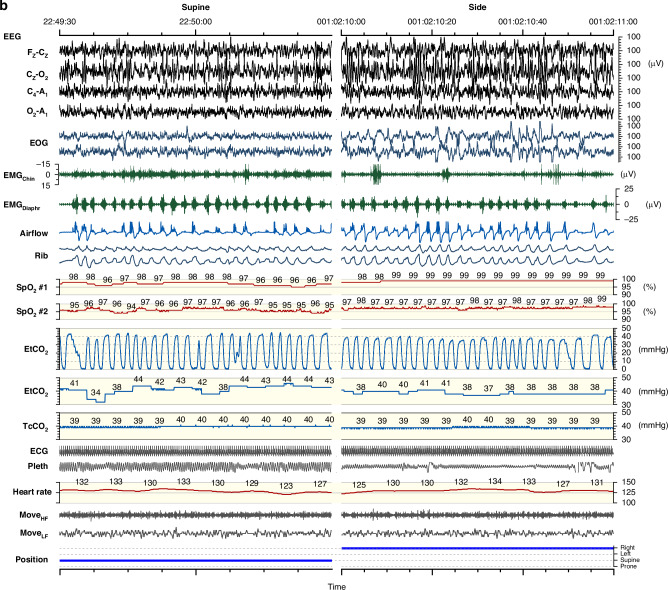
Polysomnographic (PSG) recording in a 5-month-old infant with severe laryngomalacia. **a** Summary of PSG findings, including sleep stage distribution, obstructive events, and positional dependency. Increased airway obstruction is observed in the supine position compared to the side position. **b** Raw PSG data over 1.5 min during REM sleep, comparing supine (left) and side (right) positions. In the supine position, there is more severe airflow limitation, frequent obstructive apnea and hypopneas, increased diaphragmatic EMG activity, and elevated end-tidal carbon dioxide (EtCO_2_) levels. BP blood pressure (estimated from pulse transit time), ECG electrocardiogram, EEG electroencephalogram, EMG electromyogram, EOG electrooculogram, EtCO_2_ end-tidal carbon dioxide, MoveHF movement sensor high-frequency band representing general movements, MoveLF movement sensor low-frequency band representing respiratory movements, Pleth oximeter plethysmography, Resp_freq_ respiratory rate, Rib respiratory induction plethysmography, SpO_2_ pulse oximeter oxyhemoglobin saturation, SpO₂ Desat SpO_2_ desaturation ≥3%, TcCO_2_ transcutaneous carbon dioxide.

The PSG studies (Fig. 1) included continuous monitoring of four electroencephalogram channels (Cz-Fz, Cz-O2, C4-M1, and O2-M1), two electro-oculography channels, chin and diaphragm electromyography (EMG), nasal airflow (pressure transducer), respiratory movements (thoracic and abdominal inductance plethysmography), electrocardiography, two pulse oximetry measurements for oxyhemoglobin saturation (SpO_2_) performed in 2–4-s signal averaging (Embla or SOMNO^TM^ HD, and Masimo Radical Pulse CO-Oximeter, Masimo Co, CA), end-tidal carbon dioxide (EtCO_2_) (CAP10 Capnograph, Medlab medizinische Diagnosegeräte GmbH, Germany), transcutaneous carbon dioxide (TcCO_2_) (SenTec Inc., MO), a movement-sensor mattress (Emfit Ltd, Finland), a position sensor, and synchronized video recordings. For PSG, the Embla N700 (Natus Medical Inc., WI) PSG system was used prior to 2018, and SOMNO^TM^ HD (SOMNOmedics GmbH, Germany) from September 2018 onwards. We did not use a thermistor in our PSG setting.

All PSG recordings were reanalyzed and scored (T.K.) using Embla® RemLogic™ (Natus) or SOMNOmedics DOMINO software (SOMNOmedics) together with extensive additional special-purpose software, especially to allow more detailed SpO_2_, EtCO_2_, TcCO_2_, and respiratory rate analyses. The sleep stage and respiratory event analyses we performed visually by applying AASM guidelines.^11^ Central, obstructive, mixed apneas, and obstructive hypopneas we recognized based on airflow, diaphragm EMG activity, and respiratory movements. Work of breathing (WOB) we estimated from diaphragm EMG activity, and graded as low, medium, and high EMG activity corresponding low, slightly increased, and clearly increased activity.^8^ Respiratory rate was calculated from the most reliable available signal; in principle first from abdominal inductance plethysmography but occasionally also from thoracic inductance plethysmograpy or EtCO_2_ signal if the quality of abdominal inductance plethysmography was not satisfactory.

### Estimation of laryngomalacia severity and flexible laryngeal fiberscope

We assessed the severity of laryngomalacia using scoring criteria described by Sivan and associates.^12^ The severity scoring included clinical features (history) score (maximum score 10), physical examination with clinical findings score (maximum score 4), and laryngoscopic score separating arytenoid score (maximum score 4) and epiglottic score (maximum score 4). We also classified the laryngomalacia type into three types described by Olney and associates.^13^ This classification is similar to the later described Groningen classification system.^14^

Most infants had several flexible laryngeal fiberoscopies performed during the follow-up (Table 2). For the comparison to PSG results, we used, in principle, laryngoscopy results closest to the date of performed PSG. However, we preferred the results of flexible fiberoscopy during general anesthesia [drug-induced sleep endoscopy (DISE); 15/79 (19%) infants] if available. Most flexible laryngeal fiberoscopies were performed in an upright position while awake with the application of local nasal anesthetics using Olympus ENF-V3 rhinolaryngoscope (2.6 mm outer diameter, no instrument or suction channel) by a pediatric ear-, nose- and throat (ENT) specialist. Flexible bronchoscopies were performed in the operating theater on the supine position via an endoscopy mask during sevoflurane anesthesia with spontaneous breathing in 25 infants (32%) by a pediatric surgeon or pediatric pulmonologist (T.K.). Most bronchoscopies were performed using Olympus BF-XP190 (3.1 mm outer diameter and 1.2 mm instrument or suction channel).

All available videos from outpatient clinic video laryngoscopes were re-analyzed independently by two experienced pediatric ENT specialists (M.K. and J.N.-K.). We used median values of the three different videoscores whenever video recordings were available and reanalyzed: (1) original scoring interpreted from videoscopy statement (T.K.) and (2) two separated rescores by ENT specialists.

### Statistical methods and analysis

Statistical analysis we performed with both IBM® SPSS® Statistics software version 29 and OriginPro 2024b. The PSG parameters were not normally distributed, and non-parametric tests served for the analysis. Group analyses were done with Kruskal–Wallis ANOVA or the Mann–Whitney test, and repeated measure comparisons with the Wilcoxon Signed Ranks Test. The level of significance was *p* < 0.05.

The presence of growth failure was based on the use of weight percentage. Weight percentage was calculated as the percentual change of weight against the predicted normal mean weight of infants with the same height. The normal values as based on extensive national registry data.^15^ On clinical use, the weight percentage below −15% of the predicted value indicates a clear growth failure while the percentage between −10% to −15% of the predicted value represents mild growth failure.

## Results

During the fourteen-year study period, a total of 79 PSG studies were performed in our pediatric sleep unit on infants with laryngomalacia and age less than nine months. Of these 79 infants, 69 infants were studied both in the supine and side sleeping positions allowing position comparison. Table 1 summarizes infant demographics, Table 2 flexible laryngeal fiberoscopy results and PSG characteristics, and Table 3 PSG study breathing parameter results. Of the 79 infants, 46 (58%) were male, 10 (13%) were born prematurely, and in 17 (22%) we could identify a possible co-existing predisposing factor for OSA in addition to laryngomalacia (Table 1). Most sleep studies (42/53, 79%) were performed during daytime. The median corrected age was 8 weeks [interquartile range (IQR) 5–13 weeks].

**Table 3:** Sleep and breathing events.

	All infants	Infants included in the comparison		*p* value
Parameters	Supine	Supine	Side	Supine vs side
Number of infants	77	69	69	
Sleep characteristics				
Recording time (min)	93 (60–182)	84 (60–157)	89 (61–130)	0.46
Total sleep time (TST, min)	65 (49–107)	62 (49–95)	67 (53–98)	0.42
Sleep efficiency (%)	81 (68–90)	80 (68–90)	84 (71–94)	0.27
Time in non-REM sleep (min)	44 (34–83)	42 (34–69)	45 (35–66)	0.31
Time in REM sleep (min)	22 (14–39)	21 (13–32)	23 (14–30)	0.96
Time in REM sleep / TST	29 (22–36)	29 (22–37)	30 (24–38)	0.68
Breathing characteristics				
AHI (h^−1^)	29 (16–52)	31 (18–52)	20 (8–31)	<0.0001
OAHI (h^−1^)	20.6 (8.9–48.0)	21.8 (9.9–49.7)	7.4 (1.4–25.6)	<0.0001
OAHI in REM sleep (h^−1^)	41.7 (15.3–90.2)	46.3 (16.8–100.5)	14.1 (2.5–38.8)	<0.0001
OAI (h^−1^)	4.9 (1.3–10.9)	5.6 (1.7–11.2)	1.9 (0–5.8)	<0.0001
OA average length (s)	3.3 (2.4–4.2)	3.5 (2.8–4.3)	3.5 (2.6–4.5)	0.96
OA maximum length (s)	7.0 (5.0–9.0)	7.8 (5.0–11.0)	5.9 (4.0–7.8)	0.0006
MAI (h^−1^)	1.2 (0–3.7)	1.3 (0–3.7)	0.8 (0–2.1)	0.02
MA average length (s)	5.9 (5.0–6.7)	5.8 (4.9–7.4)	5.9 (4.9–6.9)	0.98
MA maximum length (s)	7.0 (5.0–9.0)	8.0 (5.0–10.1)	7.0 (6.0–9.1)	0.69
CAI (h^−1^)	3.9 (1.5–8.1)	3.9 (1.5–7.4)	5.1 (1.7–9.7)	0.06
CAHI (h^−1^)	4.5 (2.1–9.5)	4.4 (1.9–9.5)	5.4 (2.1–10.7)	0.17
ODI_≥3_ (h^−1^)	9.0 (2.6–22.5)	9.0 (2.6–24.7)	9.7 (3.0–25.1)	0.79
ODI_≥3_ OAH (h^−1^)	2.1 (0.3–8.1)	2.2 (0.6–11.2)	1.0 (0–3.3)	0.006
ODI_≥3_ CAH (h^−1^)	1.2 (0–2.4)	1.1 (0–2.8)	2.2 (0–5.4)	0.008
SpO_2_ MinOAHI (%)	92 (88–93)	91 (88–93)	91 (86–94)	0.42
SpO_2 Median_ (%)	98 (97–99)	98 (97–99)	98 (96–98)	0.02
EtCO_2_ P_99_ (mmHg)	45.0 (41.3–48.8)	45.4 (41.3–48.8)	44.3 (39.8–48.0)	<0.0001
EtCO_2_ P_99_ in REM sleep (mmHg)	45.0 (41.3–48.8)	45.4 (41.3–49.5)	43.5 (39.8–48.0)	0.0005
TcCO_2_ P_99_ (mmHg)	44.3 (41.3–48.0)	44.6 (41.3–47.3)	44.3 (40.5–47.3)	0.41
TcCO_2_ P_99_ in REM sleep (mmHg)	43.5 (40.5–47.3)	44.3 (40.5–47.3)	42.8 (40.5–46.5)	0.73
Chin EMG activity (0–2)	0.7 (SD 0.7)	0.7 (SD 0.7)	0.4 (SD 0.6)	<0.0001
Diaphragm EMG activity (0–2)	1.3 (SD 0.7)	1.3 (SD 0.7)	0.9 (SD 0.7)	<0.0001
Breathing frequency (min^−1^)	30.0 (24.0–35.0)	30.0 (24.0–35.0)	30.0 (24.8–37.0)	0.32

Results are presented as median (IQR, interquartile range), except for chin and diaphragm EMG activity which is presented as mean (range) at a scale 0 (normal), 1 (increased), 2 (laborious). Diaphragm EMG activity was used to estimate the work of breathing.

*AHI* apnea/hypopnea index*, CA* central apnea, *CAI* central apnea index, *CAHI* central apnea and hypopnea index, *EMG* electromyography, *EtCO*_*2*_ *P*_*99*_ end-tidal carbon dioxide 99th percentile level, *MAI* mixed apnea index, *Non-REM* non-rapid eye movement, *OA* obstructive apnea, *OAHI* obstructive apnea and hypopnea index, *OAI* obstructive apnea index, *ODI*_*≥3*_ *CA* pulse oximeter desaturation index of ≥ 3% related to central apneas, *ODI*_*≥3*_ *CAH* pulse oximeter desaturation index of ≥3% related to central apneas and central hypopneas, *ODI*_*≥3*_ *OAH* pulse oximeter desaturation index of ≥3% related to obstructive and mixed apneas and obstructive hypopneas, *REM* rapid eye movement, *SpO*_*2*_ *MinOAH* pulse oximeter minimum oxyhemoglobin saturation related to obstructive and mixed apneas and obstructive hypopneas, *SpO*_*2 Median*_ pulse oximeter oxyhemoglobin saturation median value, *TcCO*_*2*_ *P*_*99*_ transcutaneous carbon dioxide 99th percentile level, *TST* total sleep time.

Comprehensive data on the correlation between clinical assessments of laryngomalacia severity and laryngoscopy findings, along with the interscorer repeatability of laryngoscopy (Supplementary Table S1), are available in the Online Data Supplement.

### Obstructive breathing in polysomnography

**Fig. 2:**
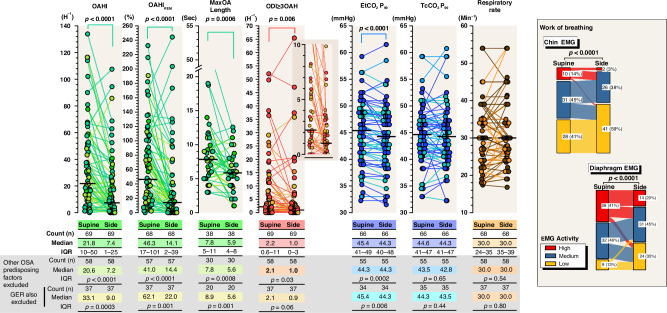
Breathing characteristics in 69 infants studied on both the supine and side sleeping positions. Number of obstructive events was higher, the length of the longest obstructive apnea was longer, a number of obstructive event-related SpO_2_ desaturations was higher, EtCO_2_ 99th percentile levels were higher, and both chin and diaphragm EMG values were higher in the supine than in the side sleeping position. The results remained essentially the same even if other known predisposing factors for OSA other than laryngomalacia were excluded such as obstructed nasal breathing, small chin, syndromes, vocal cord dysfunction, or gastroesophageal reflux (GER). EtCO_2_ P_99_ end-tidal carbon dioxide 99th percentile level, EMG electromyogram, OAHI obstructive apnea and hypopnea index, OAHI_REM_ obstructive apnea and hypopnea index in REM sleep, ODI_≥3_OAH index of oxyhemoglobin desaturations over 3% from baseline after obstructive apnea or mixed apnea or obstructive hypopnea, OSA obstructive sleep apnea, TcCO_2_ P_99_ transcutaneous carbon dioxide 99th percentile level.

Sleep study results are presented in Table 3, and in Fig. 2. Upper airway obstruction showed a clear sleep stage and sleep-position dependency (Figs. 1 and 2): obstructive apnea and hypopnea index (OAHI) was almost systematically higher in REM sleep than in non-REM-sleep and in the supine than in the side-sleeping position. In the supine position, a median OAHI was 42 h^−1^ (IQR 15–90 h^−1^) in REM-sleep and 11 h^−1^ (IQR 4–28 h^−1^) in non-REM-sleep (*p* < 0.0001). From the total sleep time, a median OAHI was 22 h^−1^ (IQR 10–50 h^−1^) in the supine position and 7 h^−1^ (IQR 1–26 h^−1^) in the side-sleeping position (Fig. 2). The sleep position dependency of OSA was also reflected in EtCO_2_ levels (*p* < 0.0001), and both chin and diaphragm EMG activity as an estimate for work of breathing (*p* < 0.0001). The results remained essentially the same even if infants with signs of other possible predisposing factors for OSA were excluded from analyses (Fig. 2, for predisposing factors for OSA see Table 1). OAHI did not correlate with the presence of additional predisposing factors.

**Fig. 3:**
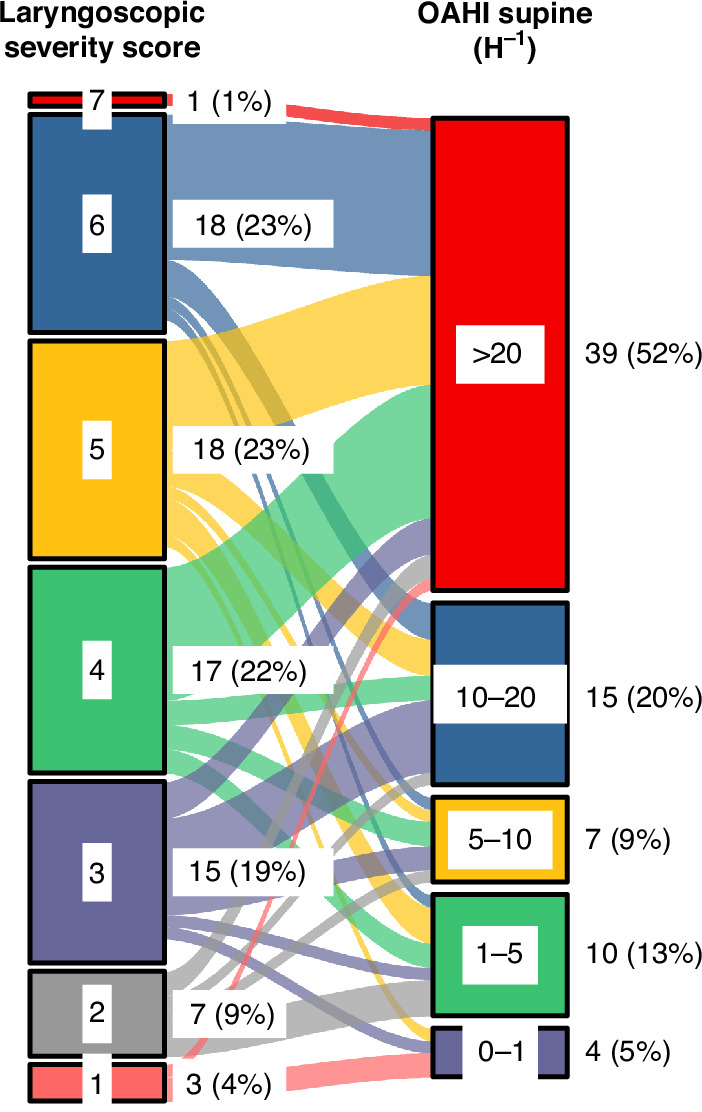
Alluvial presentation correlating severity of laryngomalacia on videolaryngoscopy and obstructive apnea and hypopnea index (OAHI). The OAHI results represent polysomnography (PSG) results on the supine sleeping position. OAHI is divided into 5 categories: 0–1 h^−1^, 1–5 h^−1^, 5–10 h^−1^, 10–20 h^−1^, and >20 h^−1^. The laryngoscopic total score did not show a clear correlation with OAHI. However, when separating arytenoid and epiglottic scores, the epiglottis score showed a weak correlation to OAHI (*R*^2^ 0.11, *p* = 0.002).

Laryngomalacia laryngoscopic score showed only a modest linear correlation to OAHI (*R*^2^ 0.10, *p* = 0.005). In addition, in automatic linear model analysis taking into account the laryngomalacia clinical score and laryngoscopic score, only the clinical score showed correlation with OAHI in the supine sleeping position (*R*^2^ 0.12, *p* = 0.001). When separating test scores into clinical features (history) score, physical examination score, arytenoid score, and epiglottis score, linear modeling showed correlation (model *R*^2^ 0.19) with both epiglottic score (predictor importance 53%, *p* = 0.04) and history score (47%, *p* = 0.006). Figure 3 shows the distribution between laryngoscopic score and OAHI.

In a rough classification of laryngomalacia into three categories presented by Olney and associates,^13,14^ Type 3 laryngomalacia or floppy epiglottis (Kruskal–Wallis ANOVA *p* = 0.002) was associated with more severe OSA and higher OAHI than Types 1 (*p* = 0.02) and 2 (*p* = 0.002) (Table 2). The response to side positioning was similar between the groups (Kruskal–Wallis ANOVA *p* = 0.49).

### Comparison to two control groups

The results of the laryngomalacia study group were compared with those of two historical study groups: infants with PRS^10^ and infants with OSA without any clear anatomical predisposing factors.^8^ Infants in the laryngomalacia group were studied, on average, at two months of age, whereas the other two groups were studied at an average age of one month (Supplementary Table S2).

OSA associated with laryngomalacia differed somewhat from the OSA observed in infants with PRS or those with OSA without any clear predisposing anatomical factors (Supplementary Table S2). Infants with PRS most frequently exhibited upper airway obstructions (OAHI), while infants with laryngomalacia showed the most laborious breathing, as indicated by the highest diaphragm EMG activity. However, the response to the alleviation of OSA when transitioning from the supine to the side sleeping position was similar across all three study groups (Supplementary Table S3).

### Growth failure

We lack appropriate information about growth in five infants. In the rest, 18 (25%) showed a clear growth failure with a weight percentage^15^ less than −20% of predicted average normal weight, and 13 (18%) infants with mild growth failure (weight between −10% to −20% of predicted normal average weight corresponding the height). In automatic linear regression model analysis taking into account of laryngomalacia clinical feature (history) score, physical examination, epiglottic score, and arytenoid score, the degree of growth and growth failure showed significant correlation only to laryngomalacia physical examination score (*R*^2^ 0.10, *p* = 0.004). From PSG parameters, in automatic linear regression model analysis taking into account OAHI, frequency of SpO_2_ desaturations (ODI3_OAH_), diaphragm EMG activity (work of breathing), and EtCO_2_ and TcCO_2_ 99th percentile values, the estimated degree of work of breathing (61%, *p* = 0.06) and EtCO2 99th percentile level (39%, *p* = 0.13) showed some tendency for weak correlation to growth failure (model *R*^2^ 0.07).

### Treatment

**Fig. 4:**
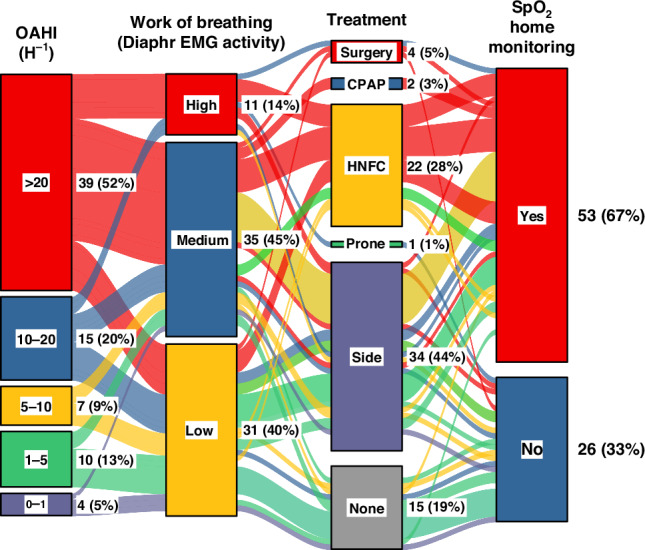
Alluvial presentation of categorial PSG findings, treatment provided, and SpO_2_ home monitoring according to the obstructive apnea and hypopnea index (OAHI) in the first polysomnography (PSG). OAHI and oxygen desaturation index (ODI_≥3_) are divided into 5 categories: 0–1 h^−1^, 1–5 h^−1^, 5–10 h^−1^, 10–20 h^−1^, and >20 h^−1^. During the first PSG, the effect of sleep position on OSA was tested in most infants. CPAP continuous positive airway pressure treatment, Diaprh diaphragm, HNFC high nasal flow cannula treatment, SpO_2_ pulse oximeter oxyhemoglobin saturation.

For PSG findings, treatment, and clinical follow-up, see the alluvial presentation in Fig. 4. After PSG, of the overall 79 infants, 4 (5%) infants had supraglottoplasty, 28 (35%) infants received positive airway pressure support either with a high-flow nasal cannula (HFNC) (26, 33%) or nasal CPAP (3, 4%). Since the OSA was almost invariably sleep-position dependent, 33 (42%) infants were treated with side-sleep positioning and sleep positioning was a part of the treatment in 56/79 (71%) of infants. HFNC treatment was accompanied by side positioning in 21/25 (84%) of cases, and by prone positioning in one infant. Home monitoring was introduced to 43/57 (75%) infants who had sleep positioning as a part of the treatment protocol.

## Discussion

We show in young infants with laryngomalacia that the appearance of upper airway obstruction is consistently sleep-stage dependent (79 infants) and frequently but not always sleep-position dependent (69 infants). Obstructive events were more common in REM- than in non-REM-sleep and breathing more laborious and high-end end-tidal carbon dioxide levels were higher on the supine than on the side sleeping position.

### Sleep position dependency of OSA

There is currently a solid literature on the appearance of OSA in infant laryngomalacia.^3–5^ However, the effect of sleep position on OSA has not been previously studied. Interestingly, OSA in young infants seems to be sleep position dependent irrespective of OSA etiology. We have applied the same clinical PSG protocol for most infant patients presenting with obstructive events since 2012. OSA in infants with PRS and in infants without any obvious structural abnormality show sleep stage and sleep position-dependent OSA in very much the same way as we noted in infants with laryngomalacia in this study.^7–10^ The main difference in laryngomalacia is the frequent presence of steady partial upper airway obstruction in non-REM sleep accompanied by increased work of breathing, and sometimes also by prolonged inspiration.

The studied laryngomalacia infant group represents infants with the severe end of laryngomalacia treated in our tertiary hospital. This is also supported by high laryngomalacia severity scores and a high prevalence of growth failure in this study cohort.

For the study purposes, we tried to identify other possible predisposing factors for OSA than laryngomalacia. In this analysis, we included small chin size, difficulty in nasal breathing, a recognized syndrome or craniofacial abnormality, or symptoms suggesting the presence of gastroesophageal reflux.^7,16^ Half of the studied infants showed signs of one or two of these predisposing factors. The position dependency of OSA severity remained the same in an infant subcohort after the exclusion of infants with these recognized other predisposing factors. In addition, OAHI did not correlate with the presence of additional predisposing factors. However, as there are no clear criteria for any of these characteristics, and none of these predisposing factors are easily quantified, we suggest that the current cohort represents any unselected cohort of infants with moderate to severe laryngomalacia.

The current study does not directly reveal any specific reason for the position dependency of OSA. The presence of OSA in laryngomalacia has been attributed to both anatomical and neurologic factors.^5,17^ Despite some differences in the upper airway anatomy, the sites of upper airway obstruction in infant OSA seem to be similar to ones observed in non-obese adults.^18^ In preschool children and adults with OSA, the change in body position from supine to side position during DISE is related to airway opening and a decrease in a tendency for airway obstruction at the tongue base and larynx.^19–22^ Similar but less effective changes have been observed in adults with head rotation only.^23,24^ We suggest that the position effect on OSA in studied infants is similar to the effect observed in older children and adults in DISE. In clinical visits, the position dependency of breathing difficulty was occasionally obvious already during wakefulness in the same way as observed in infants with PRS. This supports the idea of clear anatomical factors instead of sleep and position-related changes in the control of breathing.

According to our video analysis, the upper airway obstruction seemed to be not only position but especially head position related with relief of obstruction with head extension. We did not find any study in adults or children where the effect of head extension on upper airway collapsibility would have been studied using DISE.

In addition, the preceding awake period seemed to affect the appearance of upper airway obstruction in some of the studied infants. During a sleep period following agitation and crying, the breathing difficulty and partial upper airway obstruction may stay more severe for 5 min up to 20 min.

### Laryngoscopy

The estimation and characterization of the degree of laryngomalacia is challenging. In our study, there was a high variability between the two ENT specialists in the scoring of the degree of laryngomalacia. This was especially true in the estimation of the arytenoid score. We compared the scoring only on those cases where the laryngoscopy was performed while awake in an upright sitting position during outpatient ENT visits. Laryngoscopic studies performed in supine while asleep may have provided more consistent results.^5,12^

In clinical practice, we have noted that fiberoptic laryngoscopy findings do not necessarily have a straightforward correlation with the PSG findings and the degree of OSA. This may happen in both directions; sometimes breathing disorder on PSG appears as mild despite a severe appearance on laryngoscopy, and vice versa. This clinical notion was confirmed by our study, and the laryngoscopic score showed only a weak correlation with OAHI. Epiglottic score correlated more closely with the severity of OSA than the total laryngoscopic severity score or arytenoid score. A similar trend was also noted when following Olney and associates^13^ laryngomalacia classification, and type 3 laryngomalacia (floppy epiglottis) was associated with higher OAHI than observed in the two other laryngomalacia types, 1 and 2.

Based on our results, it seems that the laryngomalacia severity score does not predict the presence or severity of OSA and the clinical impact of scoring appears minimal. The scoring may be useful in the patient follow-up when a comparison to previous laryngoscopy findings is needed.

### Treatment and follow-up of upper airway obstruction

Our study results suggest that young infants with laryngomalacia may benefit from the side sleeping position during the first months of life, when breathing difficulty is usually at its worst. However, the natural tendency for improvement of laryngomalacia makes it difficult to evaluate the long-term benefits of any treatment. As an additional result, we have presented our treatment approach. These results are more retrospective documentation than suggestions for who to treat.

Our clinical judgment on how to treat was based on the number and degree of airway obstruction, presence and degree of SpO_2_ desaturations, estimated work of breathing, growth, and the sleep-position dependency of OSA (Figs. 1–4). We ended up recommending side-sleep positioning as the only treatment in 42% of the studied infants. Since, the OSA was position-dependent in most infants, we also applied side-sleep positioning when a more invasive HFNC or CPAP treatment was introduced, and sleep positioning was part of the treatment in the majority (71%) of infants.

Traditionally, the majority of the children with laryngomalacia are managed without any intervention.^1^ Laryngomalacia is most often self-limited with resolution of symptoms within the first two years of life.^5,6^ However, approximately 5–20% of infants with severe laryngomalacia are considered to require or benefit from surgical intervention.^1^ In our cohort, only 5% of infants had supraglottoplasty (Fig. 4) indicating some reluctance towards surgery.

Currently, the supine sleep position is recommended for all infants without any exceptions as part of SIDS‐reduction campaigns.^25^ The meta‐analysis‐based estimated odds ratio (OR) for SIDS between the supine and prone sleeping position is 4.46, and between supine and side‐sleeping positions 1.36.^26^ Single cohorts may show higher risk ratios for side positioning.^27^ We introduced home pulse oximeter monitoring for most infants when recommending side sleeping position. This approach may be criticized,^28,29^, and future studies are needed to address the usefulness of side positioning. Despite some increase in risk for SIDS, lateral or even prone positioning is widely used in the treatment of PRS in combination with home monitoring.^30,31^ HFNC treatment may alter the SIDS risk and risk for airway closure significantly.

During the study period, the importance of PSG has increased as a part of our laryngomalacia management. This has led to an increase in the importance of pediatric pulmonologists in patient evaluation, follow-up, and treatment.

### Study limitations

We included all studied infants without applying specific exclusion criteria. In the supine vs. side comparison, three infants remained who could contribute to study group heterogeneity: one with Down syndrome, one with a chromosome deletion, and one with Miller-Dieker syndrome. However, their response to OSA between supine and side sleeping positions was similar to that of the rest of the study group. Additionally, the study group is biased toward the severe end of the spectrum of infant laryngomalacia.

Although the included infants had often several flexible laryngoscopy studies during follow-up, most of these videoscopies were performed during outpatient visits in an upright position while awake (Table 2). Only one-third of the infants had drug-induced sleep endoscopy (DISE) performed. DISE is considered more reliable in laryngomalacia diagnostics than awake laryngoscopy.^12,32^ It is possible that the use of DISE in all infants could have improved the correlation between laryngoscopic severity score and PSG findings.

The majority of our PSG studies were done as daytime recordings. The recordings were approximately half the length as obtainable by standard overnight PSG. These recordings we continued as long as needed for appropriate clinical decision-making. In this age group, infants generally sleep enough during the daytime for reliable PSG analysis.^33,34^ Daytime recordings allowed a pediatric pulmonologist’s online surveillance of PSG. We thus cannot conclude that whole-night PSG recording would have significantly affected the study conclusions.

## Conclusions

In infant laryngomalacia, the degree of OSA is frequently more severe on the supine than on the side sleeping position. This applies to the number of obstructive events, work of breathing, and EtCO_2_ levels. The severity of laryngomalacia on flexible videolaryngoscopy shows only a modest correlation with the severity of OSA. In patient evaluation, clinical feature (history) score, and epiglottic score in laryngoscopy showed best correlations with the severity of OSA.

## Supplementary information

Supplementary data

## Data Availability

The datasets generated during and/or analyzed during the current study are available from the corresponding author upon reasonable request.
