# Supplementary material for: Upper airway obstruction during sleep in infants with laryngomalacia is frequently sleep-position-dependent
Source: Pediatr Res. 2025 Feb 13;98(3):1110–9. doi: 10.1038/s41390-025-03919-z (PMC12507697; doi:10.1038/s41390-025-03919-z)
Supplement: Supplementary file 1 — Supplementary data [file 41390_2025_3919_MOESM1_ESM.pdf]

1. **Title:** Upper airway obstruction during sleep in infants with laryngomalacia is frequently sleep position dependent.

2. **Authors:** Turkka Kirjavainen,[1,2,3] MD, PhD, Mervi Kanerva,[4] MD, PhD, Hanna-Leena Kukkola,[1,2] MD, Johanna Nokso-Koivisto,[4] MD, PhD

3. **Name of departments and institutions:**

   - [1]Department of Pediatrics, New Children's Hospital, Helsinki, Finland
   - [2]Pediatric Research Center, New Children's Hospital, Helsinki University Hospital, Helsinki, Finland
   - [3]Children's Hospital Department of Clinical Neurophysiology and Neurological Sciences, HUS Medical Imaging Center, Helsinki University Central Hospital, Helsinki, Finland
   - [4]Department of Otorhinolaryngology -Head and Neck Surgery, New Children's Hospital, Helsinki University Hospital, Helsinki, Finland

Supplementary data

Supplementary Table S1: Intra-scorer repeatability of the degree of laryngomalacia severity. Kappa statistics.

Supplementary Table S2: Comparison of sleep and breathing events in infants with laryngomalacia, Robin sequence, and obstructive sleep apnea (OSA) without predisposing anatomical factors.

Supplementary Table S3: Comparison of sleep and breathing events in supine and side sleeping positions across three study groups: Infants with laryngomalacia (LM), Robin sequence (R), and obstructive sleep apnea (OSA) without anatomical predisposing factors.[1]

Correlations between clinical estimation of laryngomalacia severity and laryngoscopy findings

Clinical score and laryngoscopic score showed reasonable linear correlation with R^2 0.28 ($p < 0.001$) with each other. In automatic linear regression model analysis taking into account laryngomalacia history score and physical examination, both correlated with laryngoscopic score: model R^2 0.21 ($p < 0.001$), history score with 42% proportion (p = 0.04) and physical examination with 58% ($p = 0.048$).

Repeatability of laryngoscopy analysis.

Most infants had several flexible laryngeal videoscopic studies. Laryngoscopy videos used in PSG comparisons were available for 37 children. These videos included only videolaryngoscopic studies performed while awake in upright sitting position during ENT outpatient clinic visits. The interpretation of laryngomalacia type and epiglottic scores were consistent between the original laryngoscopy statement and the two ENT specialist re-analysis. However, there was a significant variability in the arythenoid score (Friedman ANOVA $p < 0.0001$). This variability was also reflected into the variability of total severity score (Friedman ANOVA $p = 0.005$). Table S1 presents detailed data concerning interscorer repeatability and Kappa statistic.

Supplementary Table S1. Intra-scorer repeatability of the degree of laryngomalacia severity. Kappa statistics.

	Scorer		
Scoring	Scorer	ENT #1	ENT #2
Laryngomalacia score	Statement	0.24 (*p* < 0.001)	0.02 (*p* = 0.78)
	ENT #1		0.25 (*p* < 0.001)
Aryhtenoid score	Statement	0.12 (*p* = 0.13)	0.02 (*p* = 0.74)
	ENT #1		0.27 (*p* = 0.004)
Epiglottic score	Statement	0.32 (*p* < 0.001)	0.10 (*p* = 0.21)
	ENT #1		0.53 (*p* < 0.001)
LM type	Statement	-0.17 (*p* = 0.16)	0.06 (*p* = 0.68)
	ENT #1		0.01 (*p* = 0.94)

Statement origal laryngocopy statement, *ENT* pediatric ear-, nose- and throat specialists

Supplementary Table S2. Comparison of sleep and breathing events in infants with laryngomalacia (LM), Robin sequence (R),[2] and obstructive sleep apnea (OSA) without predisposing anatomical factors.[1]

	Study group			*p* value			
	Robin Sequence	OSA	Laryngomalacia	Kruskal-Wallis	Mann-Whitney		
Parameters	(R)	(O)	(LM)	ANOVA	R/LM	O/LM	R/O
Number of infants	123	91	77				
Corrected age (Weeks)	4.3 (2.9 – 7.3)	3.9 (1.7.7 – 7.3)	8.1 (4.7 – 13)	<0.0001	<0.0001	<0.0001	0.20
Sleep Characteristics							
Recording Time (Min)	257 (229 – 294)	244 (191 – 285)	228 (196 – 312)	0.06			
Total Sleep Time (TST, Min)	151 (127 – 170)	148 (121 – 177)	150 (128 – 198)	0.67			
Sleep Efficiency (%)	59 (50 – 68)	65 (55 – 71)	70 (59 – 76)	0.03	<0.0001	0.01	0.03
Time in non-REM sleep (Min)	97 (84 – 112)	95 (80 – 117)	102 (85 – 136)	0.92			
Time in REM sleep (Min)	51 (38 – 68)	48 (35 – 65)	50 (37 – 71)	0.59			
Time in REM sleep / TST	34 (28 – 41)	34 (28 – 39)	30 (24 – 38)	0.65			
Breathing Characteristics							
AHI ($Hour^{-1}$)	39 (20 – 63)	17 (9 – 28)	24 (12 – 45)	<0.0001	0.002	0.007	<0.0001
OAHI ($Hour^{-1}$)	32 (11 – 59)	7 (3 – 15)	16 (7 – 37)	<0.0001	0.0008	0.0001	<0.0001
OAHI in REM sleep ($Hour^{-1}$)	64 (23 – 123)	15 (7-27)	28 (13 – 60)	<0.0001	<0.0001	0.0007	<0.0001
OAI ($Hour^{-1}$)	17 (6 – 34)	3 (1 – 7)	4 (1 – 10)	<0.0001	<0.0001	0.23	<0.0001
OA average length (s)	4.4 (3.7 – 5.0)	4.8 (3.5 – 5.6)	3.3 (2.5 – 4.1)	0.24			
OA maximum length (s)	9.7 (7.3 – 12.5)	7.0 (5.0 – 10.0)	6.0 (4.0 – 8.4)	<0.0001	<0.0001	0.02	<0.0001
MAI ($Hour^{-1}$)	1.9 (0.8 – 3.8)	1.9 (0.9 – 3.8)	1.2 (0.4 – 2.3)	0.65			
MA average length (s)	6.9 (6.1 – 8.1)	5.8 (4.9 – 7.4)	5.9 (5.0 – 6.7)	0.50			
MA maximum length (s)	9.4 (7.2 – 12.3)	10.0 (7.0 – 13.0)	7.0 (6.0 – 9.5)	0.34			
CAI ($Hour^{-1}$)	3 (1 – 7)	7 (4 – 13)	5 (2 – 9)	<0.0001	0.03	0.003	<0.0001
$ODI_{\geq 3}$ ($Hour^{-1}$)	10 (5 – 20)	9 (4 – 21)	10 (3 – 21)	0.55			
$ODI_{\geq 3}$ OAH ($Hour^{-1}$)	3.0 (1.2 – 8.0)	1.4 (0.4 – 3.8)	1.9 (0.4 – 6.5)	0.0005	0.11	0.19	0.0005
$ODI_{\geq 3}$ CA ($Hour^{-1}$)	0.4 (0 – 1.6)	1.6 (0.4 – 3.6)	1.5 (0.3 – 3.8)	<0.0001	0.006	0.75	<0.0001
SpO_2 MinOAHI (%)	89 (86 – 91)	90 (84 – 92)	91 (86 – 94)	0.69			
$SpO_{2\,Median}$ (%)	98 (97 – 99)	98 (97 – 99)	98 (97 – 99)	0.02			
$EtCO_2$ P_{99} (mmHg)	45.0 (41.3 – 48.0)	45.0 (42.0 – 47.3)	45.0 (41.3 – 48.0)	0.81			
$EtCO_2$ P_{99} in REM sleep (mmHg)	45.0 (41.3 – 48.8)	45.0 (42.0 – 47.3)	45.0 (41.3 – 48.8)	0.77			
$TcCO_2$ P_{99} (mmHg)	48.0 (43.9 – 52.5)	45.0 (42.0 – 49.5)	45.8 (42.0 – 50.3)	0.0009	0.01	0.67	0.0009
$TcCO_2$ P_{99} in REM sleep (mmHg)	46.5 (42.8 – 51.0)	44.3 (41.3 – 47.3)	41.3 (41.3 – 48.8)	0.001	0.03	0.54	0.001
Diaphragm EMG activity (0 – 2)	0.9 (SD 0.7)	0.6 (SD 0.7)	1.3 (SD 0.7)	0.001	0.0005	<0.0001	0.001
Breathing Frequency (Min^{-1})	34 (29 – 40)	28 (26 – 34)	30 (24 – 35)	<0.0001	0.0009	0.42	<0.0001

Results are presented as median (IQR, interquartile range), except for chin and diaphragm EMG activity which is presented as mean (range) at a scale 0 (normal), 1 (increased), 2 (laborious). Diaphragm EMG activity was used to estimate work of breathing.

AHI apnea/hypopnea index, *CA* central apnea, *CAI* central apnea index, *EMG* electromyography, *$EtCO_2$ P_{99}* end-tidal carbon dioxide 99th percentile level, *MAI* mixed apnea index, *Non-REM* non-rapid eye movement, *OA* obstructive apnea, *OAHI* obstructive apnea and hypopnea index, *OAI* obstructive apnea index, *$ODI_{\geq 3}$ CA* pulse oximeter desaturation index of ≥ 3% related to central apneas, *$ODI_{\geq 3}$ OAH* pulse oximeter desaturation index of ≥ 3% related to obstructive and mixed apneas and obstructive hypopneas,

REM rapid eye movement, SpO_2 *MinOAH* pulse oximeter minimum oxyhemoglobin saturation related to obstructive and mixed apneas and obstructive hypopneas, $SpO_{2\ Median}$ pulse oximeter oxyhemoglobin saturation median value, $TcCO_2\ P_{99}$ transcutaneous carbon dioxide 99th percentile level, *TST* total sleep time

Supplementary Table S3. Comparison of sleep and breathing events in supine and side sleeping positions across three study groups: Infants with laryngomalacia (LM), Robin sequence (R),[2] and obstructive sleep apnea (OSA) without anatomical predisposing factors.[1] Comparison p-values reflect group-level differences between supine and side sleeping positions (side vs. supine).

							***p* value**			
	**Robin Sequence (R)**		**OSA (O)**		**Laryngomalacia (LM)**		**Kurskal-Wallis**	**Mann-Whitney**		
**Parameters**	**Supine**	**Side**	**Supine**	**Side**	**Supine**	**Side**	**ANOVA**	**R/LM**	**O/LM**	**R/O**
Number of infants	71	71	71	71	69	69				
Sleep Characteristics										
Recording Time (Min)	67 (48–89)	79 (61–96)	96 (66–129)	64 (42–101)	84 (60–157)	89 (61–130)	<0.0001	<0.05	0.08	<0.0001
Total Sleep Time (TST, Min)	45 (36–61)	58 (47–73)	64 (51–91)	51 (33–78)	62 (49–95)	67 (53–98)	0.0009	0.06	0.27	0.0009
Sleep Efficiency (%)	82 (57–88)	77 (64–84)	71 (56–86)	81 (66–90)	80 (68–90)	84 (71–94)	<0.05	0.47	0.33	0.05
Time in non-REM sleep (Min)	31 (26–43)	36 (27–48)	41 (34–61)	34 (29–53)	42 (34–69)	45 (35–66)	0.03	0.11	0.78	0.03
Time in REM sleep (Min)	14 (9–26)	21 (15–27)	21 (15–32)	23 (15–38)	21 (13–32)	23 (14–30)	0.13			
Time in REM sleep / TST	32 (22–46)	36 (28–47)	33 (25–40)	35 (26–49)	29 (22–37)	30 (24–38)	0.42			
Breathing Characteristics										
AHI ($Hour^{-1}$)	30 (16–68)	31 (13–68)	17 (9–34)	16 (8–26)	31 (18–52)	20 (8–31)	0.86			
OAHI ($Hour^{-1}$)	24 (9–62)	18 (4–65)	8 (5–21)	4 (0.3–10)	22 (10–50)	7 (1–26)	0.80			
OAHI in REM sleep ($Hour^{-1}$)	51 (17–131)	36 (7–82)	19 (6–37)	9 (0–21)	46 (17–101)	14 (3–39)	0.91			
OAI ($Hour^{-1}$)	12 (3–36))	9 (2–24)	4 (2–10)	0 (0–4)	6 (2–11)	2 (0–6)	0.95			
OA average length (s)	4.3 (3.6–4.9)	4.3 (3.6–5.4)	5.0 (3.8–5.8)	5.0 (4.0–6.1)	3.5 (2.8–4.3)	3.5 (2.6–4.5)	0.87			
OA maximum length (s)	7.8 (5.7–10)	7.3 (5.5–10)	8.0 (6.0–10)	7.0 (4.0–10)	7.8 (5.0–11)	5.9 (4.0–7.8)	0.20			
MAI ($Hour^{-1}$)	1.8 (0–3.8)	1.1 (0-3–0)	1.9 (0.9–3.9)	1.4 (0-4–8)	1.3 (0–3.8)	0.9 (0–2.1)	0.64			
MA average length (s)	7.4 (6.3–8.9)	7.2 (6.6–8.5)	7.2 (6.0–8.9)	7.7 (6.3–8.8)	5.8 (4.9–7.4)	5.9 (4.9–6.9)	0.81			
MA maximum length (s)	8.7 (7.2–11)	8.9 (7.0–11)	9.0 (8.0–12)	9.0 (7.0–12)	8.0 (5.0–10)	7.0 (6.0–9.1)	0.70			
CAI ($Hour^{-1}$)	2.5 (0–6.5)	3.5 (1–11)	5.6 (1.9–11)	8.5 (1.4–15)	3.9 (1.5–7.4)	5.1 (1.7–10)	0.99			
$ODI_{\geq 3}$ ($Hour^{-1}$)	7.4 (2.7–20)	12 (4–25)	6.0 (2.6–21)	12 (2.7–23)	9.0 (2.6–25)	9.7 (3.0–25)	0.31			
$ODI_{\geq 3}$ OAH ($Hour^{-1}$)	2.8 (0–6.3)	1.7 (0–7.9)	1.8 (0–5.4)	0 (0–2.9)	2.2 (0.6–11)	1.0 (0–3.3)	0.34			
$ODI_{\geq 3}$ CA ($Hour^{-1}$)	1.0 (0–2.7)	1.3 (0–7.3)	1.1 (0–2.7)	1.6 (0–4.9)	1.1 (0–2.7)	3.2 (0.7–5.7)	0.07			
SpO_2 MinOAHI (%)	91 (88–94)	90 (86–92)	91 (87–94)	89 (83–92)	91 (88–93)	91 (86–94)	0.97			
$SpO_{2\,Median}$ (%)	98 (97–99)	98 (97–99)	98 (97–99)	98 (96–99)	98 (97–99)	98 (96–98)	0.45			
$EtCO_2$ P_{99} (mmHg)	44 (41–48)	43 (40–46)	44 (42–47)	44 (41–47)	45 (41–49)	44 (42–47)	0.52			
$EtCO_2$ P_{99} in REM sleep (mmHg)	45 (41 –48)	43 (41–46)	45 (41–48)	43 (39–47)	45 (41–50)	44 (40–48)	0.72			
$TcCO_2$ P_{99} (mmHg)	46 (41–49)	45 (42–47)	44 (41–47)	44 (40–47)	45 (41–47)	44 (41–47)	0.89			
$TcCO_2$ P_{99} in REM sleep (mmHg)	44 (41–48)	44 (41–47)	44 (40–45)	43 (40–47)	44 (41–47)	43 (41–47)	0.62			
Diaphragm EMG activity (0 – 2)	0.8 (SD 0.8)	0.5 (SD 0.7)	0.7 (SD 0.7)	0.5 (SD 0.7)	1.3 (SD 0.7)	0.9 (SD 0.7)	0.81			
Breathing Frequency (Min^{-1})	32 (28–38)	32 (28–39)	28 (26–35)	30 (24–26)	30 (24–35)	30 (25–37)	0.44			

Results are presented as median (IQR, interquartile range), except for diaphragm EMG activity which is presented as mean (range) at a scale 0 (normal), 1 (increased), 2 (laborious). Diaphragm EMG activity was used to estimate work of breathing.

AHI apnea/hypopnea index, *CA* central apnea, *CAI* central apnea index, *EMG* electromyography, *$EtCO_2$ P_{99}* end-tidal carbon dioxide 99th percentile level, *MAI* mixed apnea index, *Non-REM* non-rapid eye movement, *OA* obstructive apnea, *OAHI* obstructive apnea and hypopnea index, *OAI* obstructive apnea index, *$ODI_{\geq 3}$ CA* pulse oximeter desaturation index of ≥ 3% related to central apneas, *$ODI_{\geq 3}$ OAH* pulse oximeter desaturation index of ≥ 3% related to obstructive and mixed apneas and obstructive hypopneas, *REM* rapid eye movement, *SpO_2 MinOAH* pulse oximeter minimum oxyhemoglobin saturation related to obstructive and mixed apneas and obstructive hypopneas, *$SpO_{2\ Median}$* pulse oximeter oxyhemoglobin saturation median value, *$TcCO_2$ P_{99}* transcutaneous carbon dioxide 99th percentile level, *TST* total sleep time

REFERENCES:

1. Kukkola HL, Kirjavainen T. Obstructive sleep apnea is position dependent in young infants. *Pediatr Res* 2023;93(5):1361-67. doi: 10.1038/s41390-022-02202-9 [published Online First: 20220816]
2. Kirjavainen T, Vuola P, Suominen J, Saarikko A. Micrognathia and cleft palate as a cause of obstructive sleep apnoea in infants. *Acta Paediatr* 2024 doi: 10.1111/apa.17540 [published Online First: 20241205]
